# Evaluating the Mechanical Response of Agarose-Xanthan Mixture Gels Using Tensile Testing, Numerical Simulation, and a Large Amplitude Oscillatory Shear (LAOS) Approach

**DOI:** 10.3390/foods11244042

**Published:** 2022-12-14

**Authors:** Hwabin Jung, Timilehin Martins Oyinloye, Won Byong Yoon

**Affiliations:** 1 Department of Food Science and Biotechnology, College of Agriculture and Life Sciences, Kangwon National University, Chuncheon 24341, Republic of Korea; 2 Elderly-Friendly Food Research Center, Agriculture and Life Science Research Institute, Kangwon National University, Chuncheon 24341, Republic of Korea

**Keywords:** agarose-xanthan gel system, hydrocolloid, ring tensile test, digital image analysis, finite element method (FEM) simulation, large amplitude oscillatory shear (LAOS)

## Abstract

Large deformation stress response characteristics of hydrocolloid mixture gel systems were investigated based on texture and rheological measurements. Agarose and xanthan mixtures at different ratios (1:0, 0.75:0.25, and 0.5:0.5) were chosen as the model systems. A decrease in failure stress from 2.65 to 1.82 MPa and an increase in failure strain from 0.08 to 0.13 with higher xanthan ratios were obtained based on the ring tensile test, indicating that xanthan molecules could improve the flexibility of the agarose network. The gels showed severe water loss by compression, particularly for the pure agarose gel (6.74%). Compared to the compression test, the gels presented low water loss after the ring tensile test (<1.3%) indicating that the ring tensile test could calculate the correct stress–strain relationship. Digital image correlation (DIC) and numerical simulation revealed that agarose-xanthan gel systems possess a deformation behavior with homogeneous strain distribution before failure. Elastic and viscous Lissajous–Bowditch curves from the large amplitude oscillatory shear (LAOS) measurement at different strains and frequencies elucidated that the agarose-xanthan gel was dominated by the agarose structure with a similar magnitude of elasticity at a low frequency. The large deformation approach from this study has great potential for elucidating and understanding the structure of food and biopolymer gels.

## 1. Introduction

Due to the expansion of the utilization of biopolymer gels in numerous industry fields, the demand for knowledge of physicochemical properties and principles for designing new gels is increasing. Using a combination of hydrocolloids is a way to grow the application range by modifying gel properties (e.g., texture, releasing/entrapping functional compounds, and adsorption/absorption properties) [1,2,3]. In particular, the food industry and research have a special interest in the role of hydrocolloids in the 3D-printing of food materials [4], meat analogs [5], and fat replacers [6]. In terms of the food applications for gel mixtures, textural and rheological characteristics are the most important aspects because these attributes are closely related to the mouth feel.

The mechanical response of the food materials, including food gels, is important because it affects processing, handling, and consumption. Texture profile analysis, dynamic rheology tests, and puncture or compression tests are the most common methods used to evaluate the mechanical properties of food gels [7,8,9]. However, there is still a lack of established methods to evaluate the changes in gel mechanical properties by the addition of ingredients or making binary or ternary gels. Measuring large deformation characteristics of food gels is of interest in terms of mastering food gel textures and extracting structural information about the network [10]. In particular, large deformation governs the textural response regarding the initial breakdown mechanics, which is correlated to the first bite and comminution [11,12].

Agarose is a major gelling constituent of agar which is widely used in hydrocolloid-based gels as a gelling agent and separation medium. It has a well-defined structure consisting of two alternating galactose units, 1,3-linked *β*-D-galactopyranose and 1,4-linked 3,6-anhydro-*α*-L-galactopyranose [13]. During gelation, agarose chains can aggregate into thick bundles by hydrogen bonding. The elasticity of agarose increases at higher concentrations (larger numbers of cross-links) due to an increase in the size of the junction zone. Thus, the texture becomes hard and brittle which greatly limits their applications. To overcome this drawback, agarose gels are blended with another hydrocolloid (xanthan, guar gum, konjac, etc.) or sugar (sucrose, fructose, etc.) which have been introduced in previous studies [1,14,15,16]. Therefore, mechanical and rheological characteristics can be clearly explained using the agarose mixture gels as model systems.

The present study evaluated the agarose-xanthan model system using a new approach that combined mechanical tests with image analysis, numerical simulation, and nonlinear rheological analysis. The objectives of this study were: (1) to adopt the ring tensile test to test the agarose-xanthan mixture gels to obtain the correct stress–strain relationship, (2) to examine the strain distribution of the agarose-xanthan gels and apply the obtained mechanical characteristics to the computer simulation, and (3) to validate the large deformation mechanical response based on the gel structure through LAOS measurement.

## 2. Materials and Methods

### 2.1. Sample Preparation

A 1.5% (*w/w*) agarose (A2929, Sigma-Aldrich, St. Louise, MO, USA) solution was prepared using distilled water in an Erlenmeyer flask containing a magnetic stir bar. Agarose was then dispersed on a magnetic stir plate (KR/SP46900, Johnsam Corp., Boocheon, South Korea) at room temperature overnight. The dispersion was heated in a 95 °C water bath for 2 h to melt the agarose. For the agarose-xanthan gel system, a 1.5% (*w/w*) xanthan solution prepared by dispersing the powder in distilled water at room temperature for 24 h was mixed with the hot agarose solution at different agarose-xanthan (A:X) ratios (1:1, 0.75:0.25, and 0.5:0.5) and stirred for 30 min at 90 °C. The ratio of agarose and xanthan was determined based on a preliminary experiment in which well-presented changes in gel characteristics with a proper gel-forming ability were observed. The hot solution was poured into molds for making specimens for mechanical tests or placed on a Peltier plate for dynamic rheological measurement. These molded samples were cooled down at 4 °C for 1 h and then used for experiments after temperature equilibration at room temperature (25 ± 2 °C) for 30 min.

### 2.2. Mechanical Analysis

#### 2.2.1. Uniaxial Compression Test

The compression test was conducted at room temperature (25 ± 2 °C) using a TA-XT Plus texture analyzer (Stable Micro systems Ltd., Surrey, UK) with an attached cylindrical probe (25.4 mm in diameter). The cylindrical gel with a diameter of 25.4 mm and a height of 20 mm was compressed with 80% strain at a test speed of 1 mm/s. The gel samples were placed on two layers of Whatman No. 1 filter paper (GE Healthcare Life Science, Buckinghamshire, UK) when the compression test was carried out. The weight of the filter papers was measured before and after the compression test to obtain exuded water from the gels. The measurement was conducted for at least five samples. Stress and strain curves were obtained from the compressional force and displacement:

(1) $$\sigma_{c}=\frac{F_{1}}{A}$$

(2) $$\varepsilon_{c}=\frac{\Delta L}{L}$$

where *σ_c_* is the stress, *F*_1_ is the compressional force, *A* is the compressional area, *ε_c_* is the strain, ∆*L* is the reduction in sample length, and *L* is the original length of the sample.

#### 2.2.2. Ring Tensile Test

**Figure 1:**
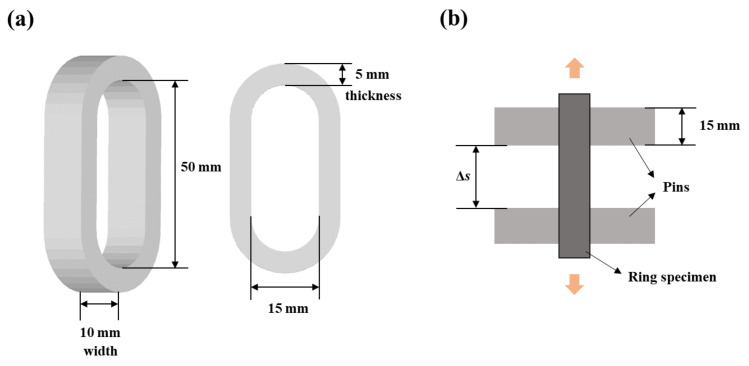
Dimensions of the ring specimen (**a**) and a schematic drawing of the front view of the ring tensile test (**b**).

The ring tensile test was carried out according to the method of Park and Yoon [17]. The oval-shaped ring sample specimen molded using sample solutions was inserted between two pins of the tensile test assembly attached to a texture analyzer (TA-XT Plus, Stable Micro Systems Ltd., Surrey, UK). A random black speckle pattern for the digital image correlation (DIC) analysis was applied to the front side of the specimen manually using a water-soluble pen prior to testing. When the measurement started, the top pin moved in a uniaxial direction at a constant speed (1 mm/s) until sample failure. At least five repetitions were conducted for this measurement. Water exudation from the ring tensile specimen was obtained by measuring the sample weight before and after the tensile test. Water on the surface was carefully wiped prior to weighing the samples. Further, videos were recorded at frame rates of 50 fps and a resolution of 0.95 million pixels using a camera (DSLR-500D, Canon Inc., Tokyo, Japan) with an 18–55 mm lens (EF-S 18–55 mm f/3.5–5.6, Canon Inc., Tokyo, Japan) during the measurement from the horizontal position of the sample to capture the displacement of each point of the speckle pattern. The front/side viewed area and height during elongation were also calculated by pixel counting using ImageJ software (National Institutes of Health, Bethesda, MD, USA) to check width and thickness changes until failure. Changes in width and thickness for all samples were small enough (< 0.5 mm) to be ignored from the calculation during the measurement. The ring tensile test setup and the ring specimen with their dimensions are illustrated in Figure 1.

Instantaneous inside circumference, inside diameter, and sample width changes during tensile elongation were evaluated as follows:

(3) $$C_{i}=d\left(\pi +2\right)+2\Delta s$$

(4) $$D_{i}=\frac{C_{i}}{\pi }$$

where *C_i_* is the instantaneous inside circumference, *d* is the diameter of the pin, ∆*s* is the elongation distance, and *D_i_* is the instantaneous inside diameter of the ring specimen.

Ring tensile stress and strain of the gel during tensile elongation considering changes in dimensions caused by large deformation were expressed according to Laterreur et al. [18] and Park and Yoon [17]. Ring tensile stress was estimated based on Laplace’s law and circumferential stress as follows:

(5) $$T=S_{c}T_{r}=\frac{F_{2}}{2wT_{r}}\times T_{r}=\frac{\sigma_{r}D_{i}}{2}$$

(6) $$\sigma_{r}=\frac{F_{2}}{wD_{i}}$$

where *T* is the wall tension by unit width, *S_c_* is the wall circumferential stress, *T_r_* is the thickness of the ring specimen, *w* is the initial sample width, *σ_r_* is the ring tensile stress, and *F*_2_ is the force measured during the ring tensile test.

The ring tensile strain was evaluated using the stretch circumference:

(7) $$\varepsilon_{r}=\frac{C_{i}}{C_{0}}$$

where *ε_r_* is the ring tensile strain and *C*_0_ is the initial inside circumference.

The ring tensile stress and strain were calculated using Equations (6) and (7) according to engineering practice. The stress–strain relationship is generally described based on the true stress and strain in a numerical simulation [18]. The ring tensile stress and strain can be converted to the true stress and strain of the uniaxial tensile test using the following equations:

(8) $$\sigma_{true}=\sigma_{r}\left(1+\varepsilon_{r}\right)$$

(9) $$\varepsilon_{true}=\ln(1+\varepsilon_{r})$$

where *σ_true_* is the true stress and *ε_true_* is the true strain.

Subsequently, the plastic region in the stress–strain curve was used to analyze elastic strain (*ε_elastic_*) and plastic strain (*ε_plastic_*) using the following equations:

(10) $$\varepsilon_{elastic}=\frac{\sigma_{true}}{E}$$

(11) $$\varepsilon_{plastic}=\varepsilon_{Total}\;-\;\varepsilon_{elastic}$$

where *ε_Total_* is the total strain and *E* is Young’s modulus. Young’s modulus is calculated from the linear region of true stress (*σ_true_*) vs. true strain (*ε_true_*).

#### 2.2.3. Digital Image Correlation (DIC)

DIC was carried out to obtain strain field during the ring tensile test by tracking speckle patterns on the deforming surface of the specimen from image frames of the video taken using a digital camera. Speckles on the specimen after deformation were compared to the reference image which had speckles on an undeformed specimen initially. Displacements were computed using pattern-matching techniques after obtaining a maximum correlation between subsets (a small area containing multiple pixels) of images before and after elongation. Two-dimensional DIC was computed using MATLAB R2022b (Mathworks^®^ Inc., Natick, MA, USA) with an open-source code (Ncorr v1.2) developed by Jones et al. [19].

### 2.3. Finite Element Method (FEM)

FEM was used to model the deformation and failure of agarose-xanthan gels during a ring tensile test. A three-dimensional model was numerically solved using the explicit dynamic module of ANSYS software (Ansys 2020 R2, Ansys Inc., Canonsburg, PA, USA). For tensile analysis, the gel specimen and the tensile apparatus were designed using ANSYS workbench 2020 R2. Dimensions used for simulation were the same as those used for experimental conditions as described in Figure 1. Geometries were discretized by constructing triangular/tetrahedral meshes. A total of 26,982 elements were generated to analyze the ring tensile test and fracture of the sample domain. True stress in the simulation was calculated using a temperature- and strain-dependent Johnson–Cook model [20,21]:

(12) $$\sigma =\left(A+B{\left(\varepsilon_{plastic}\right)}^{n}\right)\left[1+C\;ln\frac{{\dot{\varepsilon }}_{plastic}}{\varepsilon_{0}}\right]\left(1-{\left(\frac{T-T_{material}}{T_{melt}-T_{material}}\right)}^{m}\right)$$

where *σ* is the true stress, *A* is the initial yield strength of the sample at room temperature, *B* is the hardening modulus, *ε_plastic_* is the true plastic strain, *n* is the strain-hardening exponent, *C* is the parameter representing strain rate sensitivity, $\dot{\varepsilon }$*_plastic_* is the true plastic strain rate, *ε*_0_ is the reference strain rate, *m* is the parameter for the thermal softening effect, *T* is the temperature, *T_melt_* is the melting temperature, and *T_material_* is the material transition temperature.

The effect of a change in temperature by frictional reaction between the tensile apparatus and the gel sample can be neglected. Thus, the general form of the model becomes [22]:

(13) $$\sigma =\left(A+B{\left(\varepsilon_{plastic}\right)}^{n}\right)\left[1+C\;ln\frac{{\dot{\varepsilon }}_{plastic}}{\varepsilon_{0}}\right]$$

The Johnson–Cook failure model based on plastic strain was used to characterize material properties degraded by elongation during the ring tensile test:

(14) $$D=\int \frac{1}{\varepsilon_{f}}d\varepsilon_{plastic}$$

where *ε_f_* is the true fracture strain. In this model, failure took place when *D* reached a value of unity. The true fracture strain is:

(15) $$\varepsilon_{f}=\left(d_{1}+d_{2}exp^{-d_{3}\frac{\sigma_{m}}{\sigma }}\right)\left[1+d_{4}ln\frac{{\dot{\varepsilon }}_{plastic}}{\varepsilon_{0}}\right]\left(1+d_{5}\right)$$

where *d*_1_ to *d*_5_ are material damage constants obtained by the curve fitting tool in MATLAB, and *σ_m_* is the mean stress.

Boundary conditions were set with the bottom pin in a fixed position and the top pin moved along the z-axis at a rate of 1 mm/s. Material properties and Johnson–Cook parameters used for the simulation are presented in Table 1.

### 2.4. Large Amplitude Oscillatory Shear (LAOS)

The LAOS experiment was conducted on a Discovery Hybrid Rheometer (DHR-3) (TA Instruments Inc., New Castle, DE, USA) using parallel plate geometry (40 mm diameter). Coarse-grit (50 grit) sandpaper was adhered to the parallel plate and the Peltier plate to prevent sample slip. The hot agarose solution was placed between preheated plates at 70 °C with a 1 mm gap and then quenched to 4 °C to form a gel. The gel was equilibrated at 4 °C for 1 h and then at 25 °C for 30 min before starting the experiment. The sample was covered with a solvent trap to avoid water evaporation during measurements.

Strain sweeps were conducted in a range of 1 to 100% at a 1 Hz frequency to check the linear viscoelastic region (LVR) of the agarose-xanthan gels. Strain sweeps for LAOS experiments were carried out at predetermined strains from linear to nonlinear regions with frequencies of 1 and 10 Hz at 25 °C. All obtained waveform data were analyzed using TRIOS 5.1.1 software (TA Instruments Inc., New Castle, DE, USA).

### 2.5. Statistical Analysis

An analysis of variance (ANOVA) was conducted to evaluate the significant differences (*p* < 0.05) among the results followed by Tukey’s multiple comparison test. A significant difference test was performed using IBM SPSS Statistics 21 software (IBM Corp., New York, NY, USA).

## 3. Results and Discussion

### 3.1. Stress-Strain Relationships of Agarose-Xanthan Gel Systems

**Figure 2:**
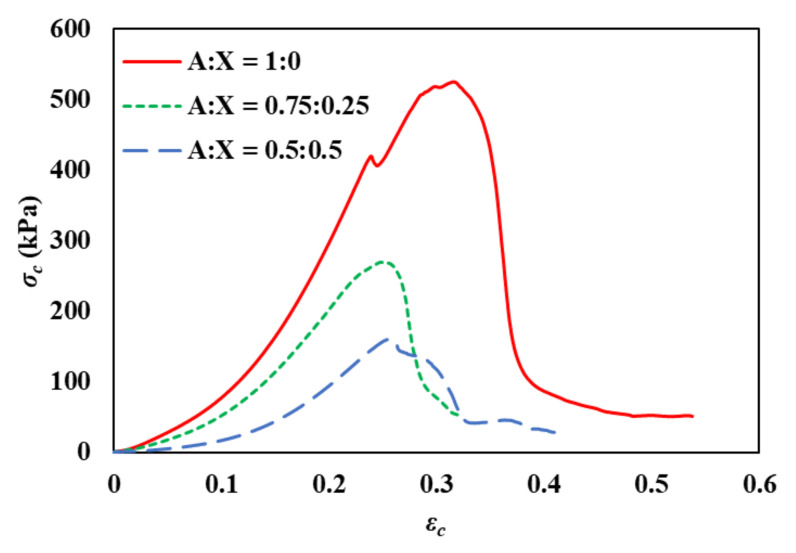
Representative stress-strain curves obtained by compression test for agarose-xanthan gel systems. A is agarose and X is xanthan.

Stress-strain curves from uniaxial compression measurements are presented in Figure 2. To discuss structure breakage by compression, fracture and failure were defined as ‘a crack propagation by structure deformation’ and ‘a complete sample separating into two or more fragments’, respectively, in our study. All samples showed failure in the strain range of 0.2–0.4 for compression measurements. However, the observation of provoking fracture was not identical to the failure point (maximum stress at the first peak) since the fracture gradually occurred from the internal structure of the sample during compression, which resulted in a broad peak of stress-strain curves around failure. The stress at failure was decreased with an increase in the xanthan ratio in the gel system. However, the fracture strain was not proportional to the ratio of agarose to xanthan (Table 2). Furthermore, all agarose-xanthan gels showed a strain-hardening behavior presenting an increase in the slope of the stress-strain curve with increasing compressive strain. It has been reported that the strain-hardening/stiffening characteristic of agarose gels is due to the semiflexible nature of agarose fibrils [23]. This result postulates that the mechanical properties of agarose-xanthan gels are dominated by the structure formed by agarose.

**Figure 3:**
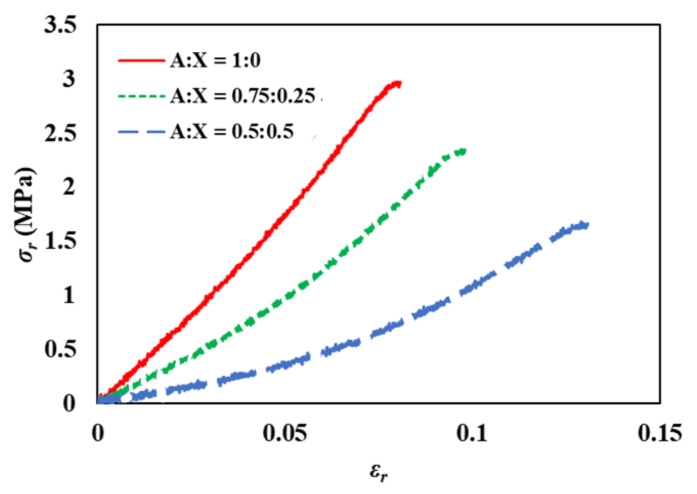
Stress-strain curves obtained by ring tensile test for agarose-xanthan gel systems. A is agarose and X is xanthan.

For results obtained from the ring tensile measurement calculated by Equations (5)–(7) (Figure 3), known to be advantageous for analyzing the stress-strain relationships of gels, the mean of failure stress was decreased when the ratio of xanthan was increased, consistent with the results of the compression test. However, the failure strain presented a great difference in that the gel with the highest xanthan ratio showed a contradictory result from the compression test (Table 2). Also, the failure point was clearer compared to the results of the compression test (Figure 3). Such differences in results between the two stress-strain measurements might have arisen from agarose-xanthan gel characteristics when responding to a compression force. There were noteworthy observations during the compression measurement, including the following: (i) a severe water exudation taking place for the pure agarose sample (A:X = 1:0) by compression (agarose-xanthan mixtures showed no visible water exudation), and (ii) high plasticity of soft agarose-xanthan gels which compelled the sample broken at the compression site. Thus, the stress of the whole specimen was hard to consider. The pure agarose gel contained 98.5% of water in this study and the water existed in the pores of the matrix mostly via capillary forces. The compression can build up hydraulic pressure registered as normal stress or shear stress [24,25]. Meanwhile, water exudation was rarely observed from gels during the ring tensile measurement which could be attributed to the deformation until sample failure for this method being relatively low compared to that in the compression test. The percentage of water loss from the gels by exudation during measurement is shown in Table 2. Water exudation by the compression test was more intense compared to that by the ring tensile test for all samples. The pure agarose gel lost approximately 7% of water by compression, whereas an increase in the xanthan ratio remarkably decreased the water loss from the gels. Water loss of the pure agarose gel by the tensile test was even lower than the compressed sample with a ratio of 0.5:0.5. In addition, there was no considerable difference in water loss among the samples for the tensile test. Thus, the stress-strain relationship obtained by the ring tensile test is more accurate and suitable for representing material properties.

The results for the ring tensile stress-strain relationship represent the effect of xanthan addition better than the compression test. Agarose-xanthan gels show lower stress and higher strain at failure compared to pure agarose gels indicating that the gels become soft and flexible with the addition of xanthan. In addition, the stress-strain curve of the pure agarose gel was close to linear. The strain-hardening phenomenon then gradually developed with an increase in the xanthan ratio. This demonstrates that pure agarose gels are relatively stiff with a stronger network compared to agarose-xanthan mixture gels. These results indicate that xanthan can restrict a strong network formation of agarose [1,15,26]. Xanthan is a stiff rod-like polysaccharide that is highly negatively charged. It is normally used as a thickener because it can form a viscous solution in cold water. Xanthan forms a jammed structure by electric repulsion. This structure shows independence from the temperature in a range of temperatures for agarose sol-gel transition [15,26]. The existence of xanthan rods hinders the formation of helices of agarose chains and the subsequent aggregation of helices into thick bundles. Therefore, agarose can form a more flexible and deformable network due to the lower aggregation of helices compared to the pure agarose gel. The stress-strain relationship obtained from the ring tensile test was suitable for investigating gel characteristics associated with its structure.

### 3.2. Local Strain Evaluated by Digital Image Correlation (DIC)

**Figure 4:**
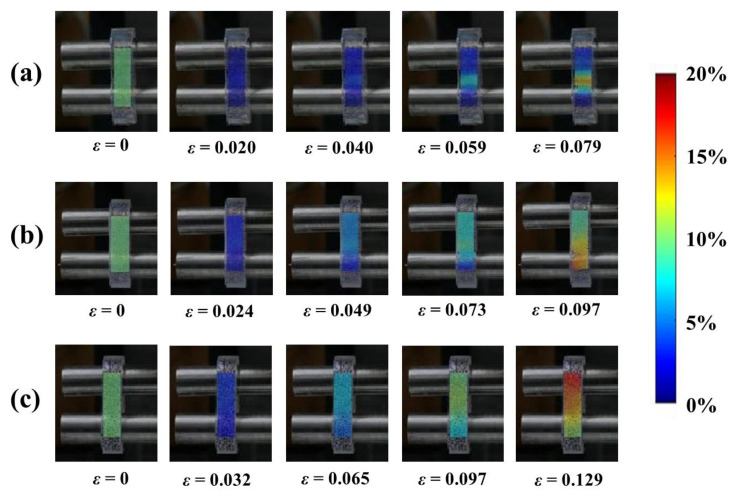
Digital image correlation (DIC) analysis of the local strain of agarose-xanthan gel systems during a ring tensile elongation until failure: (**a**) A:X = 1:0; (**b**) A:X = 0.75:0.25; (**c**) A:X = 0.5:0.5. A is agarose and X is xanthan.

Possible deficiencies in the conventional tensile test are slippage, deformation, and damage of the sample where gripped [27]. With the ring tensile test, defects arising at the grips can be avoided and stress concentration or necking can be examined by DIC analysis. Problems associated with the compression test considering the stress response from the whole specimen can be solved and proved by DIC analysis. To validate a uniform strain on the sample specimen during a ring tensile test, DIC analysis can provide information about the local strain of the sample [28]. As shown in Figure 4, all agarose-xanthan gel systems showed essentially homogeneous local strain over the entire region at an early stage of elongation. Strain concentration was then observed with larger strains at the failure location. Agarose-xanthan gel systems are highly elastic materials and, therefore, necking was not observed before failure. It should be noted here that the strain concentration observed for the pure agarose gel was due to cracking. The failure strain was low for the pure agarose gel sample and the duration between each image was relatively shorter than for other samples (~1 s). In contrast, the strain distribution at failure was less concentrated at the crack for gels containing xanthan. This result demonstrates that rod-like xanthan molecules embedded in the agarose gel can affect the flexibility of the gel network without changing the elastic characteristics of the agarose gel. The main mechanical properties of these gel systems were determined by examining the structure consisting of aggregated bundles of agarose.

### 3.3. Results of FEM Simulation

**Figure 5:**
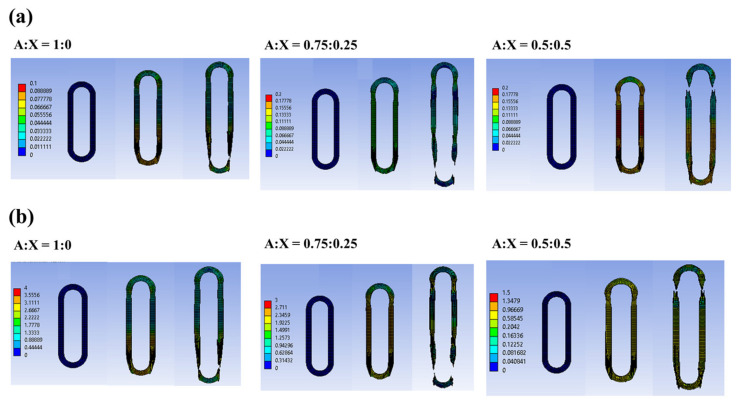
Simulation results of sample elongation and failure examined by a ring tensile test. (**a**) strain distribution, and (**b**) stress distribution in the sample during ring tensile measurement. A is agarose and X is xanthan.

Results of the numerical simulation of the ring tensile test were in agreement with the experimental results for the agarose-xanthan gel systems. Agarose gels, having higher xanthan concentrations, show lower stress but higher strain at fracture in the simulation. The material properties of the agarose-xanthan gel systems were well reflected in the simulation. As seen in Figure 5a, the tensile strain was uniformly applied through the ring sample by the elongation, and failure was observed at a random position, not around the sample holding part (pins). For the stress distribution of the sample (Figure 5b), the initial stress was developed by axial-pulling of the sample using two pins. The stress was then equally dissipated through the sample, eventually failing in a random position in agreement with Figure 5a. The simulation results well described the strain distribution obtained by DIC measurement.

### 3.4. LAOS Behaviors of Agarose-Xanthan Systems

**Figure 6:**
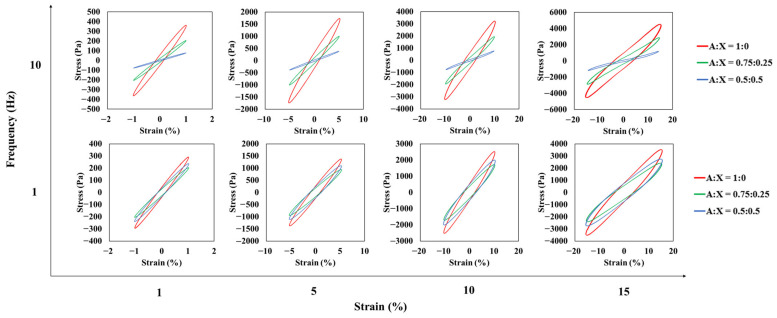
Elastic Lissajous-Bowditch curves for agarose-xanthan gel systems measured at different strains and frequencies.

**Figure 7:**
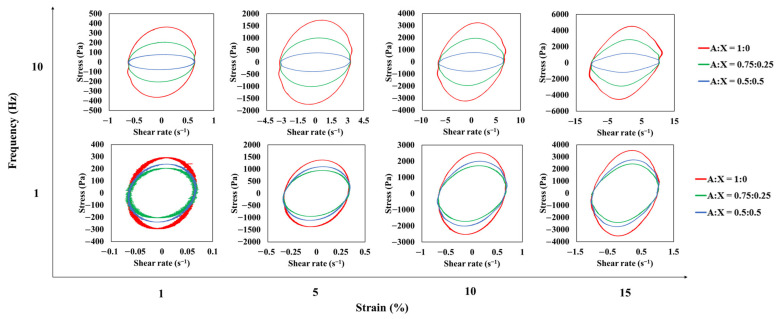
Viscous Lissajous-Bowditch curves for agarose-xanthan gel systems measured at different strains and frequencies.

The LAOS test was carried out to explain the differences in structural flexibility of the agarose-xanthan gel systems. The strain range for the LAOS measurement was decided by the strain sweep to observe both linear and nonlinear behaviors of three agarose-xanthan gel systems. Elastic and viscous Lissajous–Bowditch curves of raw stress waveforms obtained at different strains and frequencies are shown in Figure 6 and Figure 7. Within the LVR (1% strain), the Lissajous-Bowditch curves of all agarose-xanthan samples had a narrow ellipse shape for elastic curves and a circle shape for viscous curves at all frequencies, implying that these gels were elastic with nearly no contribution of viscous stress. Both elastic and viscous Lissajous–Bowditch curves beyond the LVR became distorted ellipses with an increase in strain, indicating viscous dissipation by structure breakdown. Distortions increased at a higher frequency (10 Hz) due to the LVR narrowing with subsequent decreases in the critical stress and strain with an increase in frequency [29,30]. Also, the shape of distortion described a strain-hardening behavior of agarose-xanthan gels under large deformation which was in agreement with the results of ring tensile measurements. The magnitude of the Lissajous-Bowditch curves showed no big difference among agarose-xanthan gel systems at 1 Hz. On the other hand, they became distinct at 10 Hz. This result indicated that a similar network structure might have been formed by the agarose in a relaxed state, whereas the strong network of agarose chain bundles contributed to the structure in a short relaxation time. This is a consistent result since higher fracture stress is expected for a higher concentration of agarose in the agarose-xanthan gel system. In addition, the wider curve for the pure agarose gel at 10 Hz frequency represents the higher viscous dissipation by greater structural damage, demonstrating lower flexibility [31]. The large deformation characteristics acquired by the LAOS test well supported the stress-strain relationship obtained by the ring tensile test, and captured the mechanical response originated by its structural characteristics.

## 4. Conclusions

The mechanical response of agarose-xanthan model systems under large deformation was evaluated by mechanical assessment, FEM simulation, and LAOS measurements. By comparing the two texture measurement methods, compression and ring tensile, it was proved that the results of the ring tensile measurement suitably reflect texture changes in the agarose-xanthan mixture gels. The decrease in failure stress from 2.65 to 1.82 MPa and the increase in failure strain from 0.08 to 0.13 indicate an increase in softness and flexibility. The lower water loss after texture measurement (~1.0%) than the compression test (maximum ~6.7%) shows the reliability of the ring tensile test. A complicated stress calculation for compression considering hydraulic pressure inside a sample can be circumvented. In addition, a uniform local strain and no stress concentration during the ring tensile test were confirmed by DIC analysis and simulation. Lissajous–Bowditch curves obtained by the LAOS measurement revealed stress responses of those gels at different relaxation timescales. The formation of a flexible network by the addition of xanthan was confirmed by larger strains in shorter relaxation times. The interpretation of mechanical response must be carefully carried out using techniques that can explain its origins. In these terms, the approach using mechanical measurement by the ring tensile test with DIC, FEM simulation, and LAOS measurement as supportive methods which provide important structural information, has great potential for use with food and biopolymer gels.

## Figures and Tables

**Table 1:** Agarose-xanthan gel properties and Johnson–Cook parameters. A is agarose and X is xanthan.

Parameter	A:X = 1:0	A:X = 0.75:0.25	A:X = 0.5:0.5
Young modulus, *E* (kPa)	37,891	23,619	8388
Poisson’s ratio	0.48	0.48	0.48
Density, *ρ* (kg/cm^−3^)	1000	1000	1000
Initial yield strength, *A* (kPa)	2969	2183	683
Hardening modulus, *B* (kPa)	2054	2487	2339
Strain-hardening exponent, *n*	0.00067	0.0729	0.081
Strain rate constant, *C*	0.01	0.01	0.01
Reference strain rate, *ε*_0_ (1/s)	1	1	1
Shear modulus (kPa)	12,801	7979	2834

**Table 2:** Failure stress, failure strain, and water loss of agarose-xanthan gel systems measured by mechanical tests.

	Compression Test			Ring Tensile Test		
	Agarose:Xanthan			Agarose:Xanthan		
	1:0	0.75:0.25	0.5:0.5	1:0	0.75:0.25	0.5:0.5
Failure stress (MPa)	0.69 ± 0.08 ^a^	0.29 ± 0.04 ^b^	0.15 ± 0.03 ^c^	2.65 ± 0.58 ^a^	1.97 ± 0.38 ^a^	1.82 ± 0.24 ^a^
Failure strain	0.33 ± 0.01 ^a^	0.26 ± 0.02 ^b^	0.25 ± 0.02 ^b^	0.08 ± 0.01 ^b^	0.09 ± 0.01 ^b^	0.13 ± 0.01 ^a^
Water loss (%)	6.74 ± 0.68 ^a^	2.64 ± 0.36 ^b^	1.81 ± 0.48 ^b^	1.27 ± 0.26 ^a^	0.87 ± 0.17 ^a^	1.01 ± 0.13 ^a^

Means in the same row of each test with different letters (^a–c^) are significantly different (*p* < 0.05, ANOVA).

## Data Availability

The data presented in this study are available on request from the corresponding author.
